# Earlywood Anatomy Highlights the Prevalent Role of Winter Conditions on Radial Growth of Oak at Its Distribution Boundary in NW Iberia

**DOI:** 10.3390/plants12051185

**Published:** 2023-03-06

**Authors:** Ignacio García-González, Manuel Souto-Herrero

**Affiliations:** BIOAPLIC, Departamento de Botánica, EPSE, Universidade de Santiago de Compostela, Campus Terra, 27002 Lugo, Spain

**Keywords:** dendrochronology, tree ring, quantitative wood anatomy, earlywood vessels, waterlogging, distribution boundary

## Abstract

We compared climate–growth relationships (1956–2013) of two natural pedunculate oak (*Quercus robur* L.) stands with different water-holding capacities growing at the species distribution limit of the Mediterranean Region in NW Iberia. For this, tree-ring chronologies of earlywood vessel size (separating the first row from the other vessels) and latewood width were obtained. Earlywood traits were coupled to conditions during dormancy, whereby an elevated winter temperature appears to induce a high consumption of carbohydrates, resulting in smaller vessels. This effect was reinforced by waterlogging at the wettest site, whose correlation to winter precipitation was strongly negative. Soil water regimes caused differences between vessel rows, since all earlywood vessels were controlled by winter conditions at the wettest site, but only the first row at the driest one; radial increment was related to water availability during the previous rather than the current season. This confirms our initial hypothesis that oak trees near their southern distribution boundary adopt a conservative strategy, prioritizing reserve storage under limiting conditions during the growing period. We believe that wood formation is highly dependent on the balance between the previous accumulation of carbohydrates and their consumption to maintain both respiration during dormancy and early spring growth.

## 1. Introduction

Transition zones between large biogeographical boundaries are of great ecological interest, because they act as bridges for the movement of taxa during environmental changes [1]. This is the case in the Mediterranean region, where climatic predictions indicate a tendency to increase moisture deficit during the warm season, severely impacting vegetation [2]. Thus, many temperate species reach their southernmost geographical frontier in the Iberian Peninsula, being progressively replaced by more drought-tolerant species, e.g., the nemoral oaks *Quercus robur* L. and *Q. petraea* (Matt.) Liebl. [1]. In fact, future climatic models predict a considerable reduction of the area occupied by these oaks, particularly if considering habitat fragmentation [3]. Northwestern Iberia constitutes one of these boundaries, with many small patches of *Q. robur* as dominant natural forests under the Atlantic climatic regime, but progressively replaced by Mediterranean formations towards the inland due to a moderate summer drought.

Trees in boundary areas suffer from different sources of stress, and summer drought is one of the main limitations for growth, which often leads to mortality [4,5,6]. However, a higher water demand, either by rainfall reduction or the temperature/evapotranspiration increase, is not the only climatic stress trees can face in temperate regions. High amounts of precipitation associated with a relatively high winter temperature were proposed as the main cause of oak dieback in northwestern Spain [7]. Thus, oaks in this region may be suffering from a ‘double stress’, i.e., the combined effect of summer and winter conditions. As ring-porous species, oaks form large earlywood vessels for rapid growth under high water availability in spring which often remain functional for a single growing season [8], while much smaller vessels are formed during summer water shortages [9]. Since the first elements are formed before bud break [10,11], they must be supported by previously accumulated reserves [12] as a balance between carbohydrate storage in the active period and its consumption during dormancy and spring reactivation.

Tree-ring analyses are very useful to study tree responses to environmental conditions, especially if combined with variations in xylem anatomy [13]. Among them, earlywood features constitute important tools to understand the behavior of boundary sympatric oaks [14,15,16] or the role of climate on both Mediterranean [15,17,18] and mesic areas, the latter without the existence of a prevailing climatic factor [19]. Xylem architecture is directly linked to tree hydraulics [13,17], yielding a proxy of great ecophysiological relevance and often different from tree-ring width [14,19,20], so that both tree-ring compartments can be combined to assess tree performance.

Water availability is one of the most limiting factors in terrestrial ecosystems, but it is not only driven by precipitation but also controlled by evapotranspiration, being considerably dependent on temperature, vegetation cover, and soil properties. The Standardized Precipitation-Evapotranspiration Index (SPEI) considers water regimes as a function of time, i.e., the lag between the onset of water shortage and the identification of its consequences, and has been successfully used to determine the vulnerability of forests to drought stress on a global scale [21].

In this paper, we analyze the effects of climate on the wood anatomy and radial increments of *Quercus robur* growing at its southern distribution boundary to the Mediterranean region of NW Spain. For this, we compared two stands which are representative of the most common forest patches currently existing in the area, but which strongly differ in their soil water regime. We test if the climatic control of wood formation operates through two different sources of stress in the active and dormant periods. Specifically, we hypothesize that growth is constrained by (i) low water availability limiting assimilation during summer, and (ii) high winter temperature increasing respiration and reducing available carbohydrates at growth resumption.

As previous investigations in the region suggested an association between oak tree-ring features and precipitation [7,16,22], our analyses involved a site with above-average water availability (hereafter **WET**), in contrast to a more xeric stand (**DRY**). We selected a representative number of trees per site (12 and 13 respectively), measured the earlywood vessels and the radial increment for each ring, and compared the resulting tree-ring chronologies with monthly meteorological records (1956–2013). For this, we used the latewood width (**LW**) and two earlywood variables, namely, the hydraulically weighted diameter [23] of the first vessel row (***D*_H_-r1**) and the mean vessel diameter of the vessels outside this row (***mvd*-nr1**).

## 2. Results

**Figure 1:**
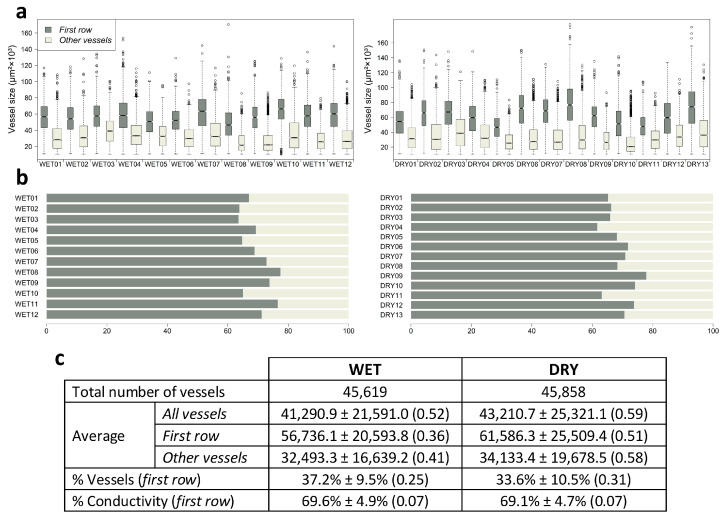
Boxplots of vessel size distribution (area in µm^2^) at each site (**a**), percentage of conductivity carried out by the first row or other vessels (**b**), and descriptive statistics (mean ± standard deviation) (**c**); values in brackets represent the coefficient of variation. Tree codes are numbered correlatively according to their site (WET or DRY).

We measured a comparable number of vessels at WET and DRY (45,619 vs. 45,858), with about one third (37.2% vs. 33.6%) belonging to the first row (Figure 1). Vessels were larger at DRY (43,210 ± 25,321 µm^2^ vs. 41,290 ± 21,591 µm^2^), which was more evident for the first row (61,586 ± 25,509 µm^2^ vs. 56,736 ± 20,594 µm^2^). However, water conductivity was mainly carried out by the first row (ca. 70%), despite its lower number of vessels, because it contained most of the largest vessels (91%).

**Figure 2:**
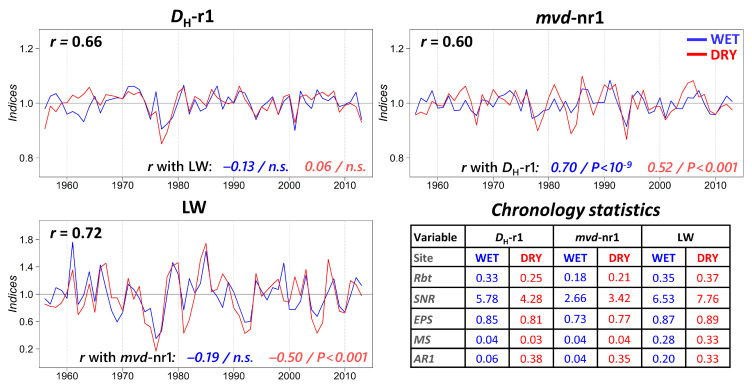
Comparison between site and variable chronologies, with their corresponding descriptive statistics, for hydraulically weighted diameter of first row vessels (*D*_H_-r1), mean vessel diameter outside that row (*mvd*-nr1), and latewood width (LW) along the common interval 1956–2013. *Rbt*: mean correlation between trees; *SNR*: signal-to-noise ratio, *EPS*: expressed population signal; *MS*: mean sensitivity; *AR1*: first order autocorrelation coefficient.

Chronologies were similar between sites but with considerable differences among variables (Figure 2). LW showed a better statistical quality than earlywood (*D*_H_-r1 and especially *mvd*-nr1), with a mean correlation higher than 0.35 and expressed population signal (EPS) greater than 0.85 in both cases, the minimum value recommended by Wigley et al. [24]. Signal-to-noise ratio (*SNR*) and *EPS* showed a similar pattern of variation. Year-to-year variability, expressed as mean sensitivity (*MS*), was very low for earlywood (0.03–0.04), and much higher for LW (0.28–0.33); in addition, LW contained abrupt growth changes that did not impact vessel size. Autocorrelation was negligible at WET, and slightly higher at DRY for *D*_H_-r1, but still with low values. As regards the differences between sites, DRY showed the best statistics for LW, whereas WET did for both earlywood variables.

Correlations between site chronologies were consideraby high (Figure 2). The common pattern was more evident for LW (*r* = 0.72) and *D*_H_-r1 (*r* = 0.66) than for *mvd*-nr1 (*r* = 0.60), which recorded the greatest differences between stands. LW was not correlated to earlywood at WET, regardless of the row considered, but *D*_H_-r1 and *mvd*-nr1 were rather similar (*r* = 0.70, *p* < 0.0001), indicating that earlywood and latewood accounted for a different kind of information. Correlation between *D*_H_-r1 and *mvd*-nr1 was also significant at DRY (*r* = 0.52, *p* < 0.0001), whereas LW was negatively correlated to *mvd*-nr1 (*r* = 0.50, *p* < 0.0001) such that smaller vessels at the end of earlywood were coupled to wider rings; similarly to WET, *D*_H_-r1 and LW were independent from each other.

**Figure 3:**
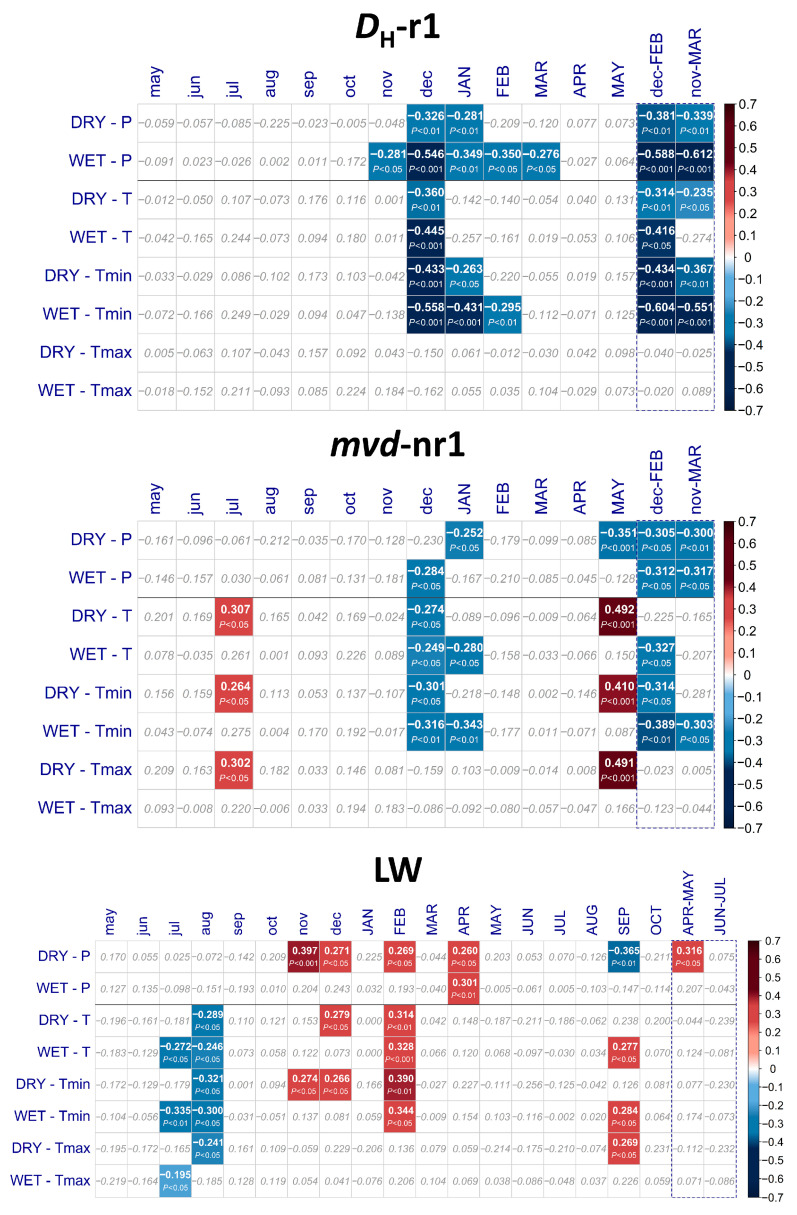
Climate–growth relationships for the earlywood (*D*_H_-r1 and *mvd*-nr1) and latewood (LW) chronologies for the period 1956–2013 (58 years); results are expressed as Pearson’s correlation coefficients, with their significance obtained by 10,000 bootstrap replications. Only significant correlations are highlighted. *D*_H_-r1: hydraulically weighted diameter of the first row; *mvd*-nr1: mean vessel diameter of vessels not belonging to the first row; LW: latewood width; *P*: total precipitation; *T*: mean temperature; *Tmin*: mean of minimum daily temperatures; *Tmax*: mean of maximum daily temperatures. Lower and uppercase letters correspond to the months of the previous and current growth years, respectively; climate variables are expressed as monthly records.

*D*_H_-r1 and *mvd*-nr1 showed strong relationships to climate (Figure 3). Differences between sites were more remarkable when comparing the pattern of responses between earlywood vessel rows.

*D*_H_-r1 had a high inverse correlation to conditions during dormancy, especially at WET (*r* = −0.612; *p* < 0.0001) for precipitation accumulated during previous November-March, and showed considerable differences to DRY, whose correlation was much lower (*r* = −0.381; *p* < 0.001, for December–February). Temperature exhibited a similar relationship, maximized by minimum temperature (*r* = −0.60, *p* < 0.0001 for WET; *r* = −0.43, *p* < 0.001 for DRY) but completely absent with maximum temperature. However, both sites exhibited a different behavior not only with regard to the role of winter precipitation, but also in the climatic response of *mvd*-nr1. While relationships at WET resembled those observed for *D*_H_-r1, albeit weaker, there was a completely different signal for DRY, as *mvd*-nr1 was positively related to temperature in May (*r* = 0.49, *p* < 0.001, for both mean and maximum temperature), i.e., a warm and/or dry spring was associated with larger vessels; a certain relation to temperature in the previous summer was also observed (*r* = 0.30, *p* < 0.05).

LW registered less significant correlations than *D*_H_ (Figure 3), evidencing a weak association to climate. Both sites had a similar pattern, with some negative relation to temperature (mean and minimum) in the previous summer (*r =* −0.27 to *r =* −0.32) and positive relation to early spring precipitation (*r* = 0.30, *p* < 0.01 at WET; *r* = 0.26, *p* < 0.05 at DRY, for April; *r* = 0.31, *p* < 0.05 at DRY, for April–May). In contrast, conditions during the end of the previous season and dormancy were more remarkable at DRY, especially for rainfall in previous November (*r* = 0.37, *p* < 0.001) and December; there was a link to conditions in February, mainly to temperature at both sites (*r* = 0.34, *p* < 0.05 at WET; *r* = 0.39, *p* < 0.01 at DRY, to minimum temperature) as well as precipitation (*r* = 0.27, *p* < 0.05 at DRY). The relationship between precipitation in September and LW (*r* = −0.37, *p* < 0.01) for DRY suggests a short-term cambial reactivation in the fall, which is also evidenced by the positive link to temperature.

**Figure 4:**
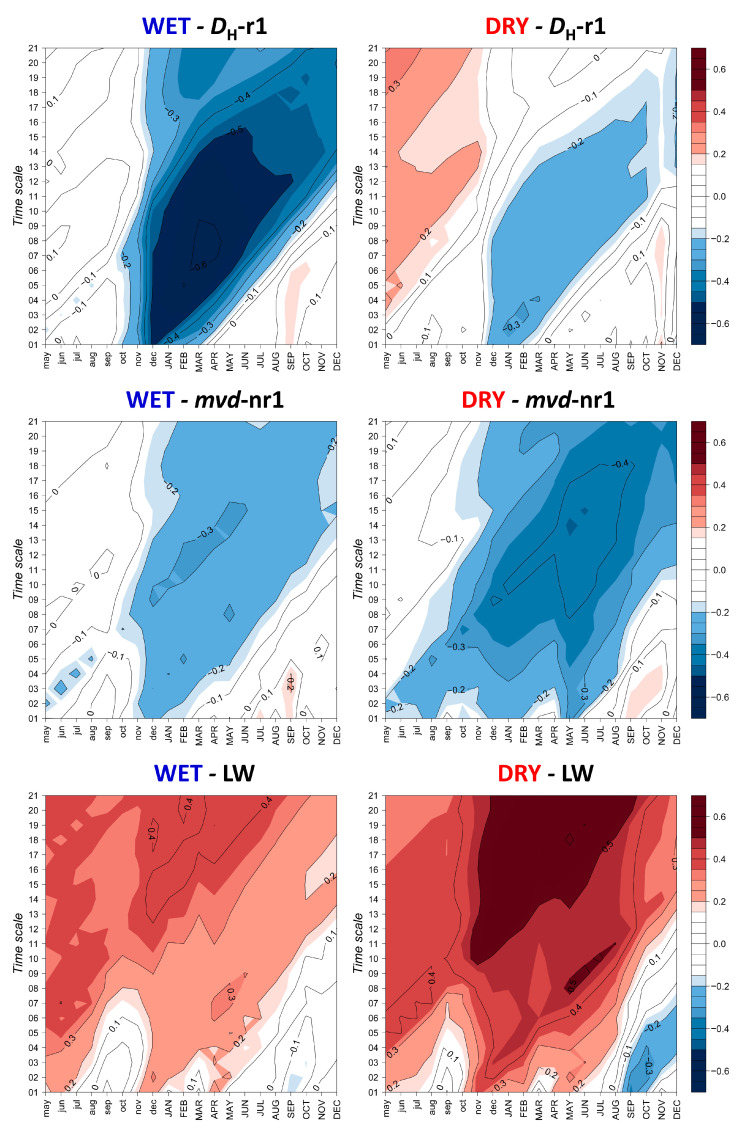
Correlations between monthly values of the standardized precipitation-evaporation index (SPEI) at different time scales (1–21) for the earlywood (*D*_H_-r1 and *mvd*-nr1) and latewood (LW) chronologies for the period 1956–2013 (58 years). *D*_H_-r1: hydraulically weighted diameter of the first row; *mvd*-nr1: mean vessel diameter of vessels not belonging to the first row; LW: latewood width. Lower and uppercase letters correspond to the months of the previous and current growth years, respectively.

Most of the differences between sites are probably due to the contrasting soil water regime, optimized when using SPEI (Figure 4), as this index indicates the magnitude and persistence of soil water excess/deficit. Thus, correlations between *D*_H_-r1/*mvd*-nr1 and SPEI highlight the differences in intensity and length of waterlogging episodes at each site. We found higher negative correlations for *D*_H_-r1 at WET, with strongly significant values at time scales of 1–20 months. The greatest influence was from previous December to current July, with a maximum in March, corresponding to a lag of six months. However, it was *mvd*-nr1 and not *D*_H_-r1 that was related to SPEI at DRY. Correlations were significant at relatively shorter time scales (8–18 months), and only for April–September, with a maximum in current May for a lag of 14 months.

The pattern was similar at both sites for LW, with a positive correlation at the same timing of the growing season, probably related to the conditions in the previous year. However, correlations were higher and spanned a longer period at DRY, reaching a maximum in May–June at lags of 18–20 months. In contrast, the highest correlation at WET was in April, with 19-month lag. The weak association with late summer at DRY was also registered for current September at a time scale of two months.

**Figure 5:**
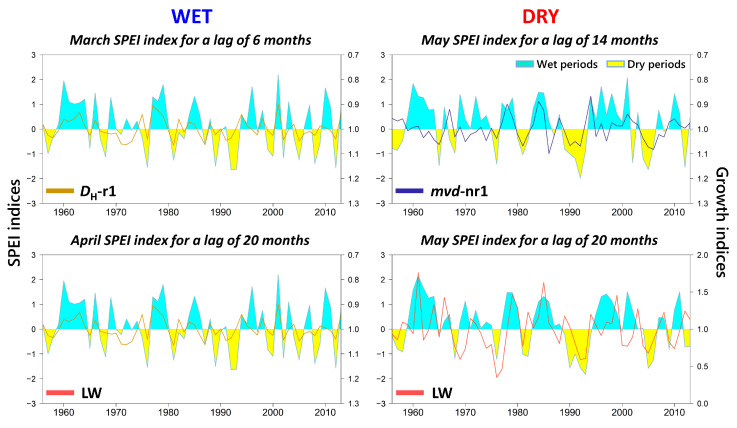
Adjustment between growth variables and SPEI values for the months and time scales maximizing correlation at both stands for the period 1956–2013 (58 years). *D*_H_-r1: hydraulically weighted diameter of the first row; *mvd*-nr1: mean vessel diameter of vessels not belonging to the first row; LW: latewood width.

The results found for the anatomical variables are supported by the adjustment between earlywood variables and monthly SPEI series maximizing correlations (March for *D*_H_-r1 at WET, and May for *mvd*-nr1 at DRY), which is consistent along the whole series (Figure 5). Similarly, this adjustment shows the clear relation between radial growth and water availability in current spring–early summer.

Finally, the divergent role of conditions during the dormant season on vessel size can also be observed in the behavior of single trees. We checked how many individuals had the same significant correlations as the site chronologies when comparing to the most relevant climatic variables (not graphically shown). For *D*_H_-r1, the relationship was much more homogeneous at WET, with 10 out of 12 trees (83%) resembling the result of the mean chronology for both temperature and precipitation in the dormant period; in contrast, only 6 out of 13 trees (46%) were related to temperature at DRY, and 8 to precipitation (62%). For *mvd*-nr1, which was linked to May conditions only at DRY, the ratio was also low (61%, i.e., 8 out of 13 trees) for both temperature and precipitation. This evidences more robust results for WET, as they are recorded not only by the mean chronology but also by most individuals.

## 3. Discussion

### 3.1. Considerations on the Selection of Variables and Site Characteristics

We studied two nearby located sites, where oaks were growing close to the southern boundary of their geographical distribution under similar climatic conditions, only differing in their soil water holding capacity. Consequently, site chronologies were highly correlated for each variable, but this similarity was highest for LW, and much lower for both *D*_H_-r1 and *mvd*-nr1. When comparing variables within sites, LW and *D*_H_-r1 were independent, LW correlated to *mvd*-nr1 only at DRY, and vessel rows were also slightly similar to each other at this site. The statistical quality of chronologies either did not differ from that successfully used for dendrochronological analysis of anatomical variables [14,17,25] or was even higher. Therefore, our results indicate that: (i) differences between both tree-ring compartments (earlywood and latewood) are remarkable; and (ii) these differences vary according to the site.

The use of earlywood anatomical variables is recommended because they usually provide another type of information than that recorded by ring width [13,16,20,26,27]. Such a detailed understanding of the climatic control of wood formation cannot be gained using ‘classical’ dendrochronology, and this is why the earlywood vessels of ring-porous trees are promising proxies in areas without a prevailing limiting factor [16,19,28]. However, the differences between both anatomical compartments are probably less under a strong limiting factor, e.g., towards xeric environments. Thus, Fonti and García-González [19] observed that the earlywood vessels of oaks had a strong climatic signal along a gradient of decreasing precipitation, but were similar to ring width for the sub-Mediterranean *Q. pubescens*, which grew under much drier conditions. Similarly, Campelo et al. [29] found a high correlation between winter––spring precipitation and both tree-ring width and maximum vessel area of the diffuse-porous *Q. ilex* growing in the southwestern Iberian Peninsula.

In view of our results, a stronger climatic control appears to be linked to more similarity among growth variables. WET is apparently the stand most limited by environmental conditions, which results in a more intense and uniform relation to climate. This explains why *mvd*-nr1 and LW were only associated at this site, and both earlywood rows were highly correlated and controlled by conditions during dormancy, whereas *mvd*-nr1 was related to late spring at DRY. Moreover, although differences between sites with regard to the overall vessel size were scarce, DRY had moderately larger vessels and more variations among individuals; however, differences were only evident for the first row. Consequently, trees at DRY were slightly more ‘ring-porous’, i.e., they exhibited more differences between the largest and the smallest earlywood vessels. Ring porosity has been described as an adaptation to temperate ecosystems with a clear contrast between seasons [9]. This is compatible with the greater range of variation in both vessel size and water availability along the season at DRY. But this idea should be considered with caution, because differences were not notable, and many other factors other than just climate could influence vessel size.

The differences between vessel rows are of methodological importance, because one of the main questions when analyzing anatomical variables is the selection among a large pool of many potential variables. Previous works with multiple variables based this decision on either an *a priori* selection, usually by means of using PCA [27,30], or *a posteriori* because of climate–growth relationships [14,26]. Nevertheless, a reduced number of variables is usually enough to understand climatic responses, provided that they account for a different kind of information [31,32]; however, splitting earlywood vessels into rows is fundamental, because vessels are formed at different times of the season, and may provide another kind of information [33]. In fact, we gained additional information by splitting vessels at DRY, but not at WET, where winter conditions were probably more limiting and the development of most vessels are consequently controlled by the same factor. We also observed that the strongest association with climate was recorded by *D*_H_-r1, an estimation of the theoretical conductivity determined by the first vessel row, which has been the preferred variable in recent papers [15,16,17,28,32]. However, other authors expressed vessel size as the mean vessel area [19,27,30]. In this case, we followed a ‘mixed’ procedure, using *D*_H_ only for the first row, but not for the other vessels, because it would give excessive importance weight to the largest vessels. This decision was based on wood formation studies in the region that showed that both groups of vessels are formed at different times [11,34,35,36]; specifically, before and after full leaf extension. As a result, conductivity is hardly dependent on the vessels formed later in the season, which are probably more important for safety. Consequently, our approach to use *D*_H_-r1 and *mvd*-nr1 separately was adequate to visualize the difference between DRY and WET, and this should be further considered in future investigations focusing on the climatic signal of earlywood vessels.

### 3.2. Effects of Climate on Wood Anatomy and Radial Increment

Earlywood vessel size was strongly linked to conditions during the dormant season at both sites, evidenced by the negative correlation with winter temperature (mean and minimum, i.e., warmer winters are coupled to smaller vessels, especially for the first row). Consequently, we hypothesize that a high winter temperature causes a considerable consumption of stored carbohydrates to maintain respiration, which in turn influences vessel size in the following spring. Furthermore, we believe that it is the dormant period, and not quiescence, that fires the response, because our study area lies at low elevation, with relatively mild winter conditions, and the correlations were maximized in December and January. The existence of an association with winter conditions was previously found for oaks in NW Iberia, not only for vessel elements [22,32], but also for tree-ring width [7,37,38]. However, a response to conditions during quiescence, and not in the dormant season, is more common for earlywood vessels [14,30].

The physiological explanation of this relationship should be linked to the oak wood anatomy. Ring-porous trees form their first vessel elements before bud break [10,11,39], and these are fundamental for tree survival because more than 90% water conductivity occurs in the earlywood of the most recent ring [40]. Consequently, they rely entirely on previous reserves to form a great part of their vascular system and photosynthetic apparatus [12,41]. Carbohydrate storage is maximum before leaf fall in autumn and minimum just after leaf expansion [42]. As carbon depletion can occur after stress episodes [43,44], we hypothesize that excessive carbohydrate consumption by a high winter respiration rate impacts earlywood formation in spring.

This explains why a concomitant high precipitation is also limiting for vessel size, a relationship maximized at WET. We are of the opinion that winter precipitation causes waterlogging at this site, firing an additional use of reserves to overcome the dormant period. Soil water saturation produces hypoxia, i.e., a low concentration of oxygen in the roots, leading to adjustments in carbon metabolism [45,46,47] which involve an anaerobic pathway and the risk of self-poisoning by fermentation end-products (ethanol). Due to the low efficiency of this fermentative pathway and the energy required to avoid cytoplasmic acidosis, the requirements to maintain the cellular energy status can be as high as 19-fold under conditions of anoxia in comparison with oxidative respiration [46]. Therefore, carbohydrate consumption at WET should be considerably increased because of its soil water regime.

These differences between sites are more remarkable for the vessels formed outside the first row, as results were contrasting. At WET, the effect of the dormant period also extended to the second and successive vessel rows, i.e., winter carbon reserves probably exert a strong control on the whole earlywood. A reliable hypothesis for this behavior could be the existence of a delay in achieving a positive balance of carbohydrates when photosynthesis is reestablished due to a low reserve availability at the moment of cambial resumption. On the contrary, vessels not in the first row were nearly unrelated to dormancy at DRY, but mostly relied on spring temperature and precipitation instead, whereby a high temperature produces a faster differentiation resulting in smaller vessels [48] The stronger limitation at WET, extending for the whole period of earlywood formation, also explains the lowest agreement between sites for *mvd*-nr1, the more remarkable differences in vessel size between rows at DRY, and the high proportion of individual trees reacting at WET. Furthermore, the use of the SPEI highlighted the differences between sites; for *D*_H_-r1, there was a strong negative effect (rainy periods) of the dormant season at WET, whereas it was much weaker and occurred later in the season for *mvd*-nr1 at DRY.

These results reinforce the evidence that conditions during dormancy are very important for the radial growth of oaks at their distribution boundary in NW Iberia. Apart from the role of winter temperature evidenced by Rozas and García-González [38], several works point out the importance of winter precipitation. Thus, González-González, Vázquez-Ruiz and García-González [22] found that vessels of *Q. robur* were limited by water excess close to a river bank, the radial increment of oaks related to both temperature and precipitation in winter in an island under warm oceanic climate [37], and Rozas and García-González [7] linked mortality oak episodes to long- and short-term periods of high winter precipitation.

We confirmed our second hypothesis that a high winter temperature was a limiting factor for cambial activity and additionally showed a detrimental effect of water excess, both apparently regulating reserve consumption during dormancy. However, our first hypothesis was based on the role of summer drought, i.e., the Mediterranean influence towards the distribution boundary of these oaks, which was more evidenced in the relationships to SPEI. A ‘classic’ dendrochronological analysis of climate–growth relationships did not show a clear effect of water availability during the growing season; only DRY had a certain positive response to precipitation during spring, which was probably lacking at WET due to the higher soil water holding capacity. In fact, the existing effect of drought upon growth was only evidenced by the SPEI, confirming its usefulness for forest ecosystems [21]; it was also not immediate but started in the previous season, while extending for a longer period. These relationships occurred for time scales of more than 20 months, were stronger, and spanned a longer period for DRY. Apparently, the most relevant role of water availability on latewood increment is not direct, but probably regulates carbohydrate dynamics. Summer water stress would limit photosynthesis and therefore reserve storage in the previous season; this is evidenced by the negative response to temperature, which indicates a high evapotranspirative demand for a species not adapted to avoid water loss, as opposed to sub-Mediterranean oaks [1]. Later on, winter water excess modulates its consumption during dormancy and growth reactivation, as observed in the earlywood.

We believe that oaks under limiting conditions in transitional areas appear to prioritize reserve storage rather than growth during the summertime. The lack of response in current summer suggests that trees slow down active radial growth in order to guarantee enough reserves for dormancy and spring reactivation. In fact, monitoring of cambial activity close to our study sites showed that wood formation there ceased earlier than in more oceanic areas [36].

## 4. Materials and Methods

### 4.1. Study Area and Sites

**Figure 6:**
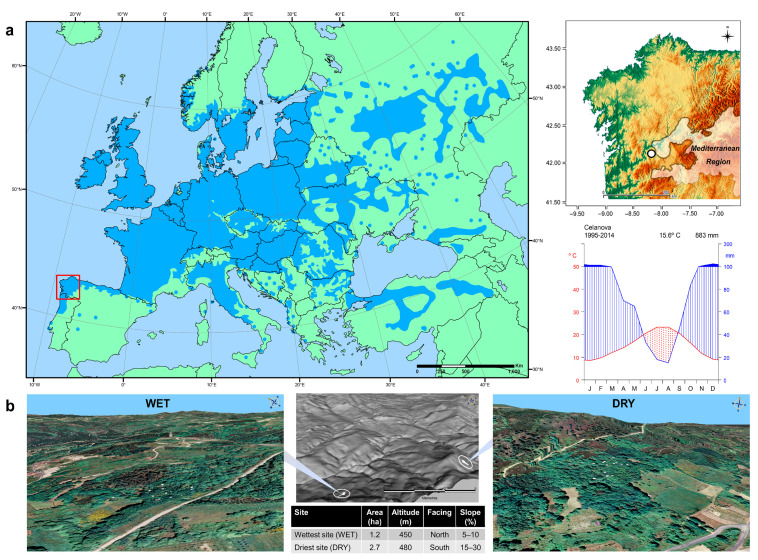
Natural distribution of *Quercus robur* (source: EUFORGEN 2009, https://www.euforgen.org/, accessed on 23 July 2015) and location of the study area at its southern range boundary in the Iberian Peninsula (**a**), showing the climate diagram from a nearby weather station. Digital Terrain Models with sampled trees as white dots and physiographic characterization of the two chronology sites are also included (**b**).

The study area lies at the southern distribution boundary of *Quercus robur* in NW Spain, on a basin ca. 500 m asl. surrounded by gentle hills not higher than 900 m (Figure 6). The climatic regime is subhumid Atlantic, with a strong seasonality. Rainfall is moderate (800 mm yr^−1^), mainly concentrated in autumn and winter, though lower than in most of the region (>1000 mm yr^−1^). Summers are warm (mean temperate higher than 20 °C in July and August) and dry, with a drought period of at least two months. Winter is relatively mild (around 7 °C in January and February), but late frosts are common due to the inland location.

We selected two nearby growing natural stands on different topographical positions. Both sites are subhumid Atlantic oak forests of the association *Rusco aculeati-Quercetum roboris* [49], dominated by *Q. robur*, with *Q. pyrenaica* present in the canopy, and lacking understory species linked to moist conditions that are common in other oak forests of NW Iberia. In contrast, Mediterranean or thermophile elements usually occur, such as *Laurus nobilis* L. or *Arbutus unedo* L., *Ruscus aculeatus* L., *Tamus communis* L.; other typical species are *Holcus mollis* L. or *Lonicera periclymenum* L.

The wettest site (WET) is a little woodland located at 450 m asl. on a gentle slope facing north, surrounded by meadows. The soil is deep, and a watercourse runs seasonally, so water availability is higher than at other stands in the area. The largest oaks are regularly distributed throughout the stand, and young *Laurus nobilis* trees commonly occur under the main canopy, with other thermophile species such as *Ruscus aculeatus* or several ferns associated with water streams. In the past, this forest served as pastureland for cattle during the summertime, as well as for fuelwood and timber extraction.

The driest site (DRY) occupies a moderate slope facing south at 480 m asl. and is derived from the abandonment of ancient farmlands. The oldest trees are abundant and regularly distributed throughout the plot, and some originate from oldest pollarded oaks that separate contiguous fields. Floristic composition is similar to WET, with the thermophile presence of *Ruscus aculeatus* as an understory species but without *Laurus nobilis*, and there is a considerable scarcity of ferns. In addition, the invasive species *Acacia dealbata* Link. occurs at the forest edge and in disturbed areas, evidencing more human pressure than at WET.

### 4.2. Processing and Measurement of Tree Rings and Earlywood Vessels

We used an increment borer at breast height and extracted two 5 mm cores from 14 dominant trees at WET and 15 at DRY. Cores were air-dried, glued onto wooden supports, and prepared for an optimal visualization of the cross-sectional surface. We obtained a regular surface by means of a WSL sliding microtome [50], followed by manual polishing with very fine sandpaper (grain sizes from P600 to P1200, Federation of European Producers of Abrasives). Wood dust and tyloses were cleaned from vessel lumina using high-pressure water blast [51]. Samples were finally stained with black printer ink, and the earlywood vessels were filled with chalk dust [14] for an optimal contrast for image analysis.

We measured earlywood width (EW) and latewood width (LW) to the nearest 0.001 mm under a binocular microscope (Olympus SZ60) at 20–40× magnification, using a tree-ring measuring linear stage (Velmex TA UniSlide, Velmex Inc., Bloomfield, NY, USA). Earlywood/latewood boundary was discriminated using vessel size and wood structure [20]; earlywood conductive elements are much larger and disposed in a continuous band, in contrast to the smaller latewood vessels in radial flamelike groups. For cross-dating, we visually compared individual curves of total ring width (RW), calculated as the sum of EW and LW, and assessed their statistical accuracy with COFECHA [52]. EW and RW series were useful for quality control of the data, but were not further used, because their information would be redundant with other variables.

We performed vessel measurements in a subset of 25 trees (12 from WET, 13 from DRY), with no breaks, anomalies, or unsure dating, which were highly correlated with the local chronology. The cores were digitized using a camera (Canon EOS 600D) coupled to the binocular microscope which automatically moves each sample along a mechanically driven platform in order to sequentially obtain high-resolution images (5184 × 3456, 17.9 Mpx) from the core surface. Pictures from the same core were stitched into a single image using PTGui ver. 9.1.8 Pro (New House Internet Services B.V., Rotterdam, The Netherlands), and saved into a Tagged Image File Format (TIFF) file to avoid loss of quality. Images were analyzed in ImageJ [53] using the VesselJ plugin (García-González, not published), automatically measuring earlywood vessels on an 8-bit (gray) threshold level due to contrast differences between the dark background tissue and the white vessel lumina. We removed objects larger than 10,000 µm^2^ or that were twice longer than the object width, whereas other undesired objects detected as vessels, or incomplete vessel outlines, had to be corrected manually. The application of the convex hull and a morphological operation (erode-dilate 2 × 2 one pass) optimized vessel shape before storing the values.

Since the whole core was analyzed as a single image, each vessel had to be assigned to the correctly dated ring after analysis. For this, we used the self-developed program Autovasos (García González, not published) to detect and modify tree-ring boundaries along with their corresponding earlywood vessels, as well as to cross-check the rings detected on the images against the dated RW series. During vessel assignment, we also separated the first row of vessels from the others [54]; all vessels located immediately after the ring boundary or initiating not farther than the imaginary line connecting the centroids of boundary vessels were considered to belong to the first row.

### 4.3. Chronology Building and Assessment

We obtained chronologies on a tree basis, i.e., we averaged width measurements and pooled all vessels belonging to the same ring but discriminated between vessels in the first row (**r1**) and those not located in this row (**nr1**), as they are formed at different moments of the growing season and potentially controlled by different driving factors. We expressed vessel size in the first row as the hydraulically weighted diameter (*D*_H_-r1), following [23], which is an estimate of the average diameter to achieve the theoretical hydraulic conductivity for a given stem. As this parameter maximizes the largest vessels, mostly located at the beginning of the ring, we preferred to express vessel size for the rest of the vessel as mean vessel diameter (*mvd*-nr1). Latewood width (LW) was also included in the analyses to better evaluate radial increment.

We fit a cubic smoothing spline (32-year cutoff, 50% variance reduction) to the series [55], which is flexible enough to remove age- and disturbance-related trends, while adapting to the ascending and descending patterns of both anatomical variables and latewood, respectively. Dimensionless growth indices [56] were computed by division and averaged into a mean chronology using a biweight robust mean [57].

We obtained six chronologies (*D*_H_-r1, *mvd*-nr1, and LW at both sites) for all subsequent analyses, along a common interval 1956–2013 (58 years) covered by at least 11 trees per site. The statistical quality of all series was assessed by descriptive statistics (mean sensitivity (*MS*) for year-to-year variability; first order autocorrelation coefficient (*AR1*) for the influence of prior growth), while the common signal of chronologies was evaluated by several coefficients commonly used in dendrochronology [55], namely, the mean correlation between trees (*Rbt*), the signal to noise ratio (*SNR*), and the expressed population signal (*EPS*). *Rbt* is the mean value of all possible Pearson’s cross-correlation coefficients; *SNR* is the resulting value from the division between *Rbt* and the statistical amount relative to noise; and *EPS* indicates the extent to which the sample size is representative of a theoretical population with an infinite number of individuals [24]. The former is independent of sample size, whereas the other two are influenced by the number of trees. We also compared variable site chronologies by their Pearson’s cross correlation.

### 4.4. Climate–Growth Relationships

We established stationary correlations between the chronologies and climatic data (meteorological records and a drought index) for 1956–2013 (58 years). Additionally, correlation functions were computed for single trees, as individual responses can provide valuable additional information [58]. We used simple Pearson’s correlations, and their significance was assessed by 10,000 bootstrap iterations, following the percentile confidence intervals. All calculations were performed in R [59], using the ‘boot’ and ‘corrplot’ packages for the scripts.

Series of monthly temperature and precipitation data were taken from the gridded data source CRU TS 3.22, available in KNMI Climate Explorer (http://climexp.knmi.nl/, downloaded on 19 February 2019) at 0.5° of spatial resolution and covering the period 1901–2013. We also calculated the Standardized Precipitation-Evapotranspiration Index (SPEI); this multiscalar index involves water balance (precipitation minus potential evapotranspiration), the accumulation of water deficit/surplus at different time scales, and the adjustment to a log-logistic probability distribution [60], and was successfully used to study responses of vegetation growth to drought [61]. Values for the index were calculated in R, using the package ‘SPEI’ and using time scales of 1 to 21 months; from this, we estimated potential evapotranspiration following the Thornthwaite method [62].

Based on previous cambial studies in the region [36], climate–growth relationships spanned the seasonal period that could apparently influence xylem formation, i.e., from previous to current May (13 months) for earlywood and to current October (18 months) for LW; relations to SPEI were analyzed until December (20 months). In addition, we seasonalized monthly values, considering dormancy–quiescence (December–February) for earlywood, and spring (April–May) and early summer (June–July) for LW.

## 5. Conclusions

Our study was able to identify the main climatic variables associated with both earlywood and latewood growth of *Q. robur* in a region located near the limit of its natural distribution boundary in southern Europe. We observed a strong link between the first earlywood vessels and conditions during winter, which was maximized under a high water-holding capacity.

Although our analysis was only based on retrospective observations of radial increments and wood anatomical features, we hypothesize that it is the balance of carbohydrates (i.e., their synthesis, accumulation, and consumption) all throughout the season that controls wood formation, rather than a direct effect of climate at the moment of cambial activity. We believe that winter modulates the reserves available for earlywood formation, but also that this importance of the dormant season is a consequence of the limiting conditions imposed by the Mediterranean influence in the active period. As carbon assimilation during the growing season is not high, trees appear to adopt a conservative strategy and prioritize reserve storage rather than radial increment.

Previous works in the region observed recent mortality at the distribution boundary of this species after extremely rainy winter periods [7]; therefore, the role of conditions during dormancy should be further explored as a stress factor, including the analysis of wood formation and carbohydrate dynamics.

## Data Availability

Data used for these analyses will be archived in Zenodo after manuscript acceptance.
